# Transgenic zebrafish model for quantification and visualization of tissue toxicity caused by alloying elements in newly developed biodegradable metal

**DOI:** 10.1038/s41598-018-32313-5

**Published:** 2018-09-14

**Authors:** Hyung-Seop Han, Gun Hyuk Jang, Indong Jun, Hyunseon Seo, Jimin Park, Sion Glyn-Jones, Hyun-Kwang Seok, Kwan Hyi Lee, Diego Mantovani, Yu-Chan Kim, James R. Edwards

**Affiliations:** 1 Botnar Research Centre, Nuffield Department of Orthopaedics, Rheumatology and Musculoskeletal Sciences, University of Oxford, Oxford, OX3 7LD UK; 2 Center for Biomaterials, Korea Institute of Science and Technology, Seoul, 02792 Republic of Korea; 3 NuclixBio, Seoul, 08380 Republic of Korea; 4 Department of Materials Science and Engineering, Massachusetts Institute of Technology, Cambridge, Massachusetts, 02139 USA; 5 Division of Bio-Medical Science and Technology, KIST School, Korea University of Science and Technology, Seoul, 02792 Republic of Korea; 6 Laboratory for Biomaterials and Bioengineering, CRC-I, Department Min-Met-Materials Engineering & CHU de Québec Research Center, Laval University, Quebec City, Canada

## Abstract

The cytotoxicity of alloying elements in newly developed biodegradable metals can be assessed through relatively low-cost and rapid *in vitro* studies using different cell types. However, such approaches have limitations; as such, additional investigations in small mammalian models are required that recapitulate the physiological environment. In this study, we established a zebrafish (*Danio rerio*) model for cytotoxicity evaluations that combines the physiological aspects of an animal model with the speed and simplicity of a cell-based assay. The model was used to assess the cytotoxicity of five common alloying elements in biodegradable implant materials. Conventional *in vitro* testing using heart, liver, and endothelial cell lines performed in parallel with zebrafish studies revealed statistically significant differences in toxicity (up to 100-fold), along with distinct changes in the morphology of the heart, liver, and blood vessels that were undetectable in cell cultures. These results indicate that our zebrafish model is a useful alternative to mammalian systems for accurately and rapidly evaluating the *in vivo* toxicity of newly developed metallic materials.

## Introduction

Biomedical implants constructed from magnesium (Mg) alloys that degrade over time have been shown to promote tissue repair and regeneration—for instance, in bone fracture healing and cardiovascular disease^1–4^. Biodegradable metallic materials provide mechanical support that allows damaged tissues to heal sufficiently, and then degrade over time as they are completely replaced by normal host tissue^5–7^. Substituting currently used inert implant materials with biodegradable alternatives eliminates the need for a second surgery to remove the implanted device, circumvents complications arising from implants that loosen with increasing age and changes in body size and shape (e.g., as a result of age-related bone loss), and reduces the socio-economic burden of an aging society on the healthcare system^8,9^.

Selection of an alloying element is a critical aspect of the material development process, as the functionality and mechanical properties of the material can be improved by modifying the type and quantity of the alloy^10,11^. Common alloying elements for Mg-based biodegradable metallic implants include biocompatible elements such as calcium (Ca) and zinc (Zn); the rare earth element yttrium (Y) (which is linked to liver toxicity) and aluminum (Al) (which is associated with Alzheimer’s disease) are also often integrated into materials to improve mechanical properties and increase corrosion resistance. As these alloys are in direct contact with living tissues, it is critical to determine the toxicity of particles released from the implant as it degrades^12–15^.

The toxicity of biodegradable materials is initially evaluated *in vitro* using different cell types treated with a metal ion in chloride form or an extract of the degrading alloy in medium^16–19^, with cell viability serving as a measure of cytotoxicity. Such assays are efficient in terms of time and cost. However, two-dimensional cell monolayers cannot recapitulate all aspects of the complex *in vivo* physiological environment. Small mammalian models are more useful in this regard for evaluating toxicity and estimating the biodegradation profile of alloying elements in humans.

**Figure 1:**
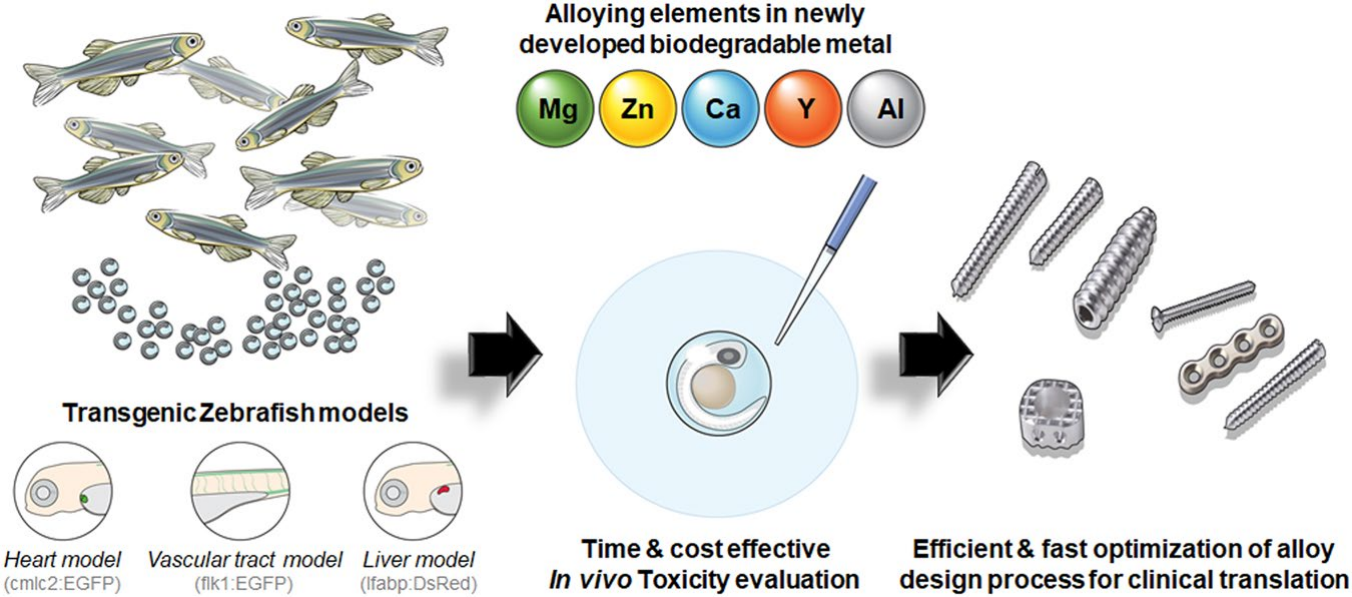
Schematic illustration of *in vivo* toxicity evaluation in zebrafish. Embryos collected from adult transgenic zebrafish were placed in a culture plate and exposed to alloying elements.

For decades, mice, rats, rabbits, and dogs have been used for toxicology experiments, but these experiments are often expensive and time consuming^20^. An *in vivo* model using zebrafish (*Danio rerio)* was recently proposed for nanotoxicity assessment^21,22^ based on the homology between fish and human genomes and anatomical and physiological similarities of the cardiovascular, nervous, and digestive systems^23–25^. Zebrafish embryos take less than 1 week to generate these major organ structures^26,27^ and remain transparent throughout development, allowing real-time observation of this process^28,29^. Additionally, with the high fecundity rate of 200–300 eggs per day every 5–7 days, the cost and time of toxicity testing are significantly reduced compared to those when using larger animals^30,31^. Thus, using a zebrafish model to evaluate the *in vivo* toxicity of degrading particles from newly developed biodegradable metal implants can accelerate the material development process to clinical application, while circumventing the ethical considerations associated with using mammals as research tools (Fig. 1).

In the present study, zebrafish embryos were treated with different concentrations of the five most commonly used elements for alloying with biodegradable metal (Mg, Zn, Ca, Y, and Al ions) to assess their toxicity. Standard cellular toxicity tests using established heart, liver, and endothelial cell lines were carried out in parallel to compare *in vitro* and *in vivo* toxicities. Transgenic zebrafish lines expressing enhanced green fluorescent protein (EGFP) or red fluorescent protein (RFP; DsRed) in the blood vessels, heart and liver, and nervous system under the control of the promoters of fetal liver kinase (flk)1, cardiac myosin light chain (cmlc)2, liver-type fatty acid-binding protein (lfabp), glial fibrillary acidic protein (GFAP), and oligodendrocyte transcription factor (olig)2 were used to visualize developmental defects in these tissues caused by the alloying ions at various concentrations.

## Results and Discussion

### Effects of MgCl_2_, ZnCl_2_, and CaCl_2_*in vitro* and in a zebrafish model

**Figure 2:**
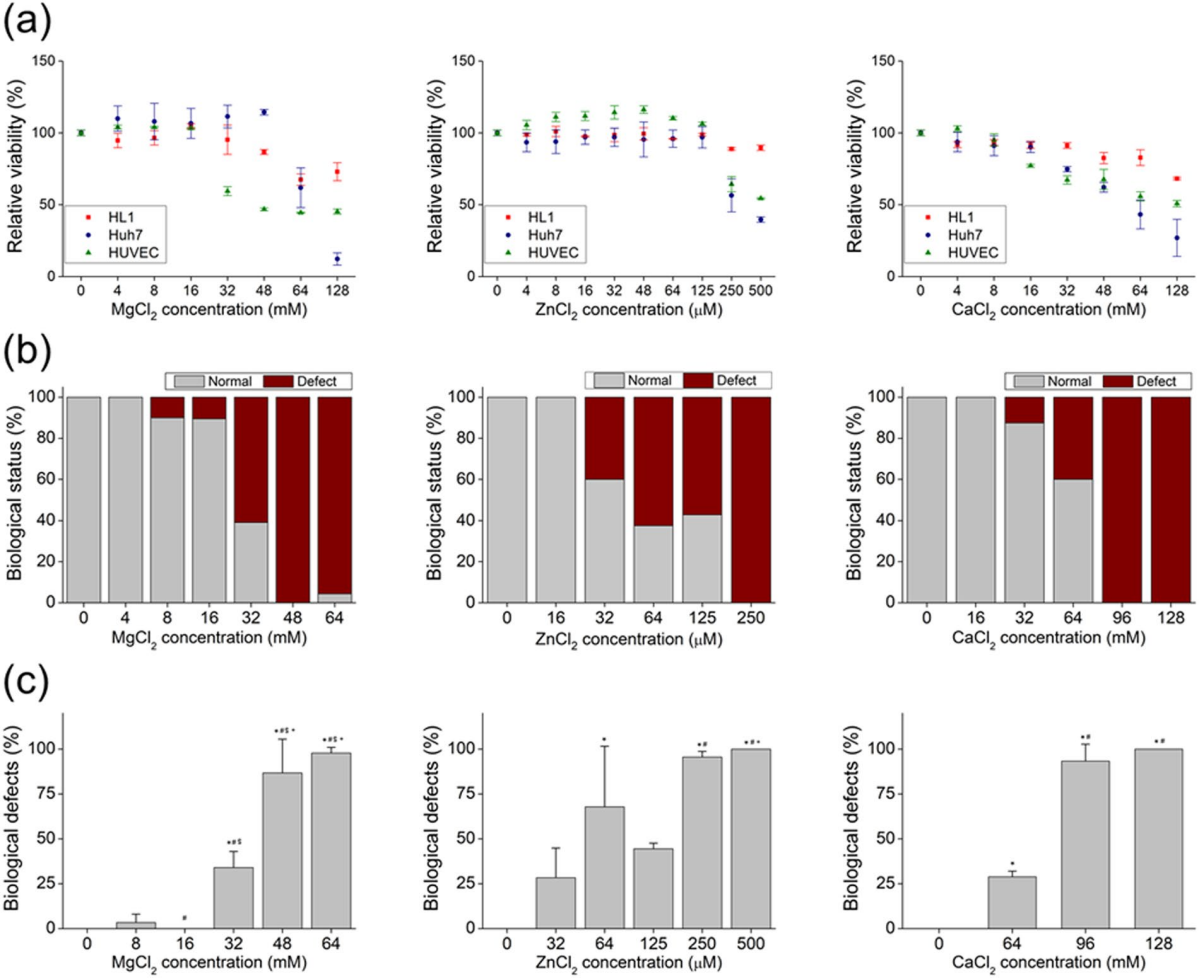
Biological effects of Mg^2+^, Zn^2+^, and Ca^2+^ *in vitro* and in a Zebrafish model. (**a**) Viability of HL-1 and Huh7 cells and HUVECs after treatment with indicated ion solutions for 12 h. (**b**) Biological status (normal or defective) of embryos at 6 hpf after treatment with indicated ion solutions. (**c**) Biological defects in embryos at 6 hpf after treatment with indicated ion solutions. *, #, &, and +, Significant difference vs. 0, 8, 16, and 32 mM Mg^2+^ ion, respectively. *, #, &, and +, Significant difference vs. 0, 32, 64, and 125 μM Zn^2+^ ion, respectively. * and #, Significant difference vs. 0 and 64 mM Ca^2+^ ion, respectively.

The viability of Huh7 hepatocytes, human umbilical vein endothelial cells (HUVECs), and HL-1 cardiac muscle-like cells treated with Mg^2+^, Zn^2+^, and Ca^2+^ for 12 h was evaluated. The viability of HL-1 and Huh7 cells was unaffected by treatment with Mg^2+^ up to 48 mM, after which viability decreased to 67.49% ± 4.00% at 64 mM and 61.80% ± 13.85% at 128 mM (Fig. 2a). HUVECs were more sensitive than the other two cell types, with a reduction in viability from 103.22% ± 0.58% to below 44.44% ± 0.34% with increasing Mg^2+^ concentration (16 to 64 mM). Treatment with Zn^2+^ had a less potent effect on the viability profiles of all cell types, which remained above the baseline value (100%) at concentrations up to 125 μM and diminished at a concentration higher than 250 μM (Fig. 2a). For example, the viability of cells exposed to 250 μM Zn was 88.87% ± 0.94%, 59.50% ± 11.52%, and 64.36% ± 5.30% for HL-1 and Huh7 cells and HUVECs, respectively. In cells treated with Ca^2+^, viability decreased at concentrations of 48, 32, and 16 mM for HL-1 and Huh7 cells and HUVECs, respectively (Fig. 2a).

To clarify the *in vivo* biological effects of Mg, Zn, and Ca ions released from biodegradable metal, zebrafish embryos were treated with increasing concentrations of MgCl_2_, ZnCl_2_, and CaCl_2_ starting 6 hours after fertilization (hpf). Unhatched eggs and dead or abnormal larvae were classified as biological defects. We initially tested the same concentration ranges as in the *in vitro* cell viability assay. Defects were observed in groups treated with 8 mM MgCl_2_ and reached 100% at concentrations of 48 mM or higher (Fig. 2b,c). In ZnCl_2_-treated groups, biological defects began to appear at 32 μM and reached 100% at concentrations greater than 250 μM. In the presence of CaCl_2_, biological defects were noted starting at 64 mM, reaching 100% at 128 mM and higher concentrations (Fig. 2b,c). These results confirm the *in vitro* findings that Mg, Zn, and Ca ions impair cell growth.

**Figure 3:**
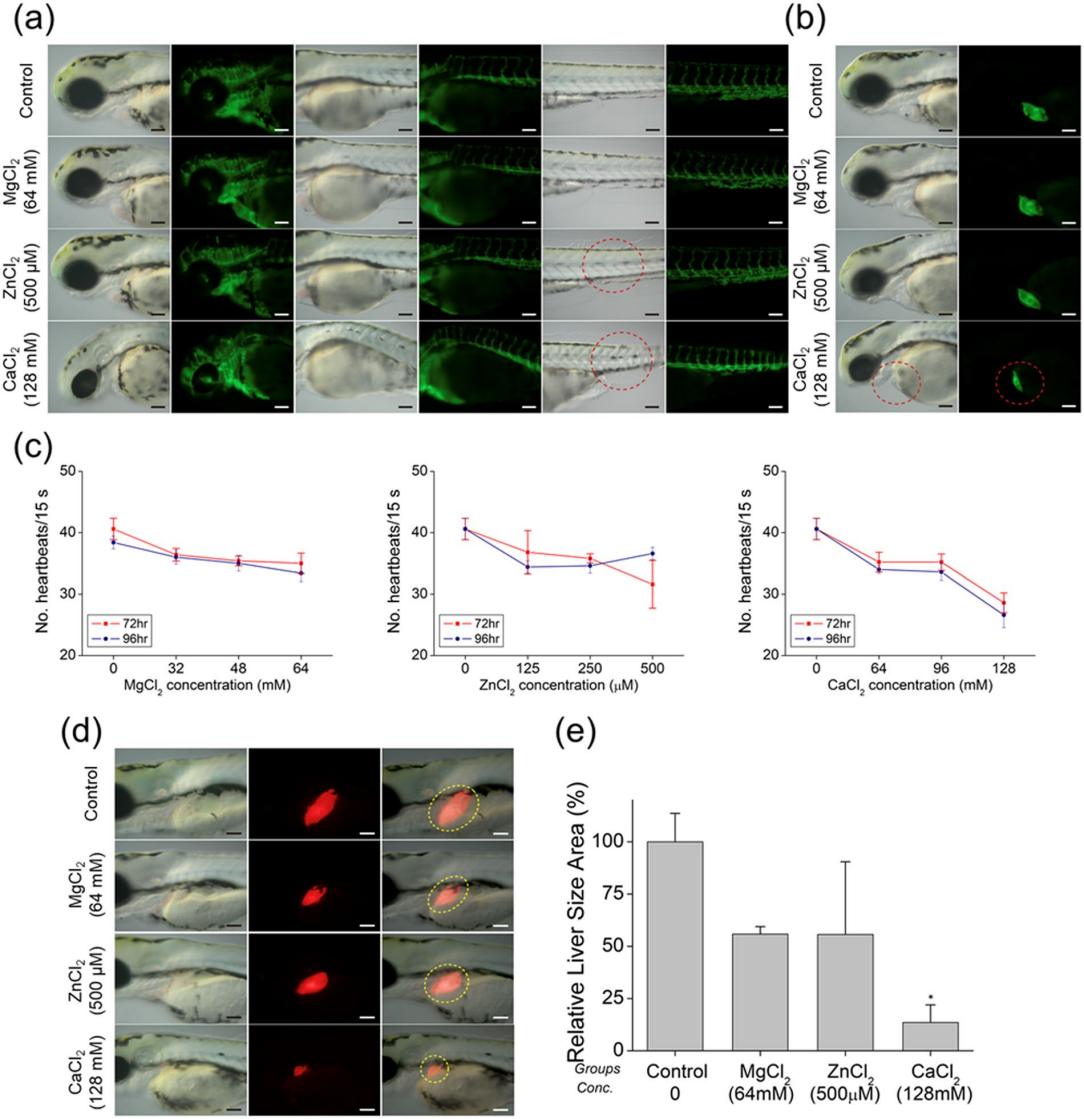
Representative images of ion-treated zebrafish embryos used to determine heart rate at (**a**) 72 and (**b**) 96 hpf. (**c**) Mean heart rate of zebrafish embryos following 72- and 96-hpf treatments with indicated concentrations of different ions (Mg^2+^, Zn^2+^ and Ca^2+^). (**d**) Representative images of ion-treated zebrafish embryos used determine liver size at 96 h. (**e**) Mean liver size of zebrafish embryos after treatment with Mg (64 mM), Zn (500 μM), and Ca (128 mM) ions. * indicates significant difference compared to controls (*p* < 0.05).

As the blood vessels and heart are among the first organs that are exposed to foreign substances during development, we evaluated embryonic defects in these tissues following exposure to alloy elements using the vasculature (Tg(flk1:EGFP)) and cardiac (Tg(cmlc2:EGFP)) transgenic zebrafish lines that express EGFP at the surface of blood vessels and cardiac tissue, respectively. We used the ion concentrations that caused biological defects in 100% of samples in the previous experiment. Embryos treated with MgCl_2_ had normal blood vessels throughout development, whereas abnormalities were observed in those treated with ZnCl_2_ and CaCl_2_ (Fig. 3a). Among Tg(cmlc2:EGFP) zebrafish embryos, only those treated with 128 mM CaCl_2_ showed abnormal heart phenotypes (Fig. 3b). The effect on cardiac function was assessed by counting the heartbeat at 72 and 96 hpf. Embryos treated with Mg, Ca, and Zn showed a decreased heart rate compared to control group embryos (Fig. 3c), particularly those treated with CaCl_2_ (Supplementary Table 2). Previous *in vitro* biocompatibility studies showed that the viability of human fetal osteoblasts treated with Mg and Zn ions was 100% at much higher doses^32^. These results demonstrate that the influx of biocompatible metallic ions into the living body affects the vascular system and causes heart function to deteriorate at a much lower dosage.

The liver is responsible for detoxification of harmful substances introduced into the body. To examine the effects of MgCl_2_, ZnCl_2_, and CaCl_2_ on liver development, we used the Tg(lfabp:DsRed) zebrafish line that expresses RFP specifically in the liver (Fig. 3d). The relative size of the liver was measured to identify any liver defects. We observed that liver size decreased by 55.82% ± 2.06%, 55.66% ± 20.07%, and 13.52% ± 4.89% at 96 hpf in the presence of MgCl_2_ (64 mM), ZnCl_2_ (500 μM), and CaCl_2_ (128 mM), respectively, compared to that of controls. This decrease was confirmed by examining Tg(lfabp:RFP) embryos treated with the various ions by fluorescence microscopy (Fig. 3e). Importantly, biological defects were induced at significantly lower concentrations than the cell viability experiments would suggest (Supplementary Table 1).

### Biological effects of YCl_3_*in vitro* and in a zebrafish model

The viability of hepatocytes and endothelial and cardiac muscle-like cell lines following exposure to increasing concentrations of Y^3+^ ion for 12 h was largely unaffected at concentrations up to 25 μM in all groups. However, there was a marked reduction in viability at 200, 50, and 100 μM for HL-1 and Huh7 cells and HUVECs, respectively.

**Figure 4:**
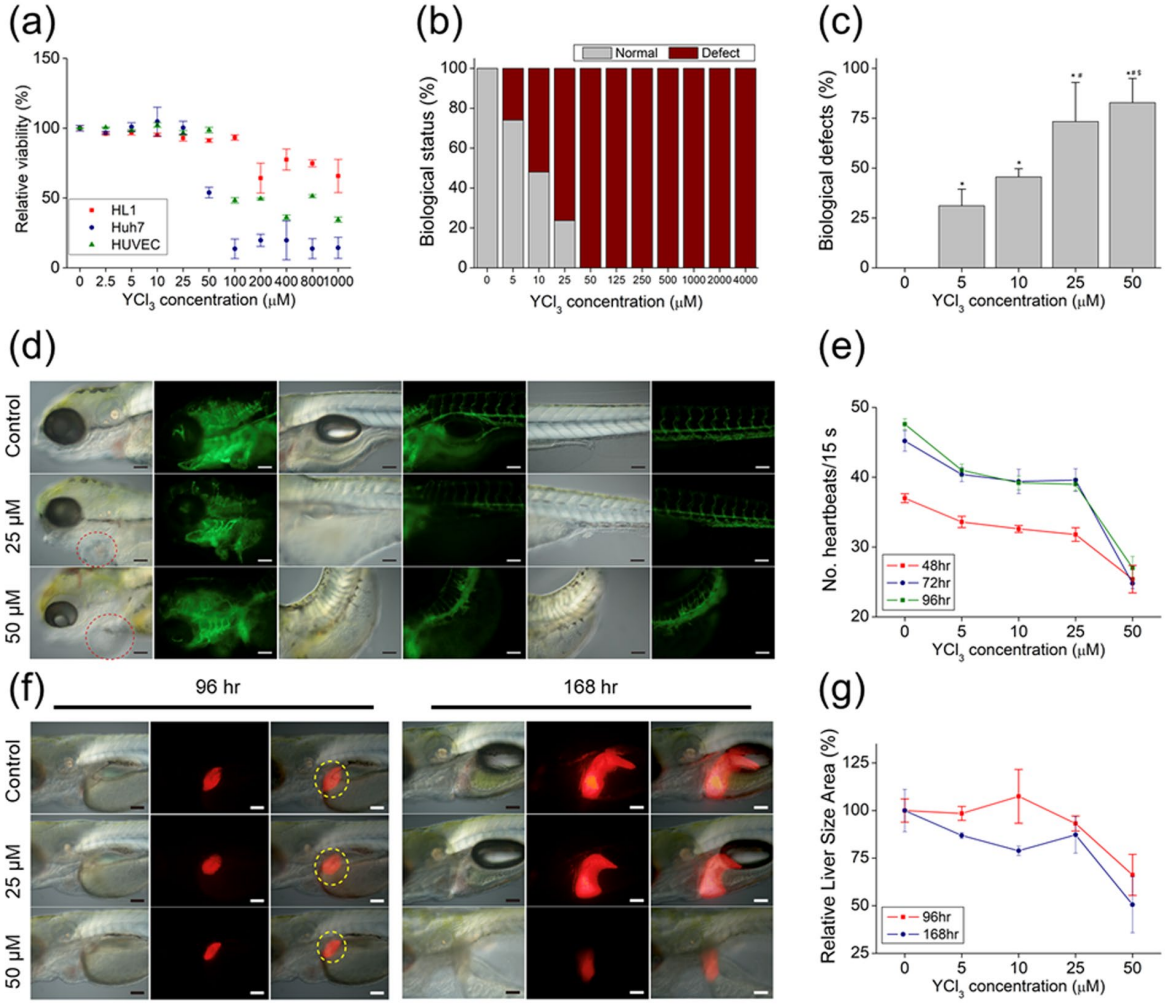
Biological effect of Y^3+^ *in vitro* and in a zebrafish model. (**a**) Viability of HL-1 and Huh7 cells and HUVECs after treatment with indicated ion solutions for 12 h. (**b**) Biological status of embryos (normal or defective) at 6 hpf after treatment with indicated ion solutions. (**c**) Biological defects in embryos at 6 hpf after treatment with indicated ion solutions. (**d**) Representative images of ion-treated zebrafish embryos used to determine heart rate. (**e**) Mean heart rate of zebrafish embryos following 48-, 72-, and 96-hpf treatments with indicated concentrations of Y^3+^. (**f**) Representative images of ion-treated zebrafish embryos used to determine liver size at 96 and 168 hpf. (**e**) Mean liver size of zebrafish embryos following 96- and 168-hpf treatment with indicated concentrations of Y^3+^. *, #, &, and +, Significant difference vs. 0, 5, and 10 μM Y^3+^ ion, respectively.

To assess the biological effect of Y released from a biodegradable Mg alloy, zebrafish embryos were treated with various concentrations of YCl_3_ (5 μM to 4 mM). Biological defects were observed in the 5 μM treatment group, and reached 100% in embryos treated with concentration higher than 50 μM (Fig. 4b,c and Supplementary Table 1).

We used the Tg(flk1:EGFP) and Tg(cmlc2:EGFP) zebrafish lines to observe the effect of Y on cardiac tissue and vessels. Embryos treated with 25 μM YCl_3_ showed heart defects, and exposure to a concentration of 50 μM resulted in cardiac edema and a bent tail (Fig. 4d). A closer examination revealed significant cardiac abnormalities, including delayed heart development and a reduced heart rate (Fig. 4e and Supplementary Movies 1 and 2). In particular, a marked decrease in heart rate—which is closely related to cardiac function—was observed in the presence of 50 μM YCl_3._ Thus, YCl_3_ introduced into a living organism at concentrations greater than 25 μM could lead to blood vessel defects, deterioration of heart function, and diminished heart rate.

There was a slight difference in liver size between Tg(lfabp:DsRed) embryos treated with 25 μM YCl_3_ and control group embryos at 96 and 168 hpf. In contrast, liver size was significantly reduced in embryos treated with 50 μM YCl_3_ to 66.20% ± 6.23% at 96 hpf and 50.58% ± 8.49% at 168 hpf. This was supported by morphological and phenotypic analyses (Fig. 4f,e), indicating that the toxic effects from Y ions are to some extent tolerated up to a concentration of 25 μM. Previous *in vitro* studies have shown that YCl_3_ is toxic at concentrations of 1 mM or higher^33^. Our *in vitro* and *in vivo* results demonstrate that YCl_3_ induces toxicity at concentrations above 50 μM.

### Biological effects of AlCl_3_ in a zebrafish model

**Figure 5:**
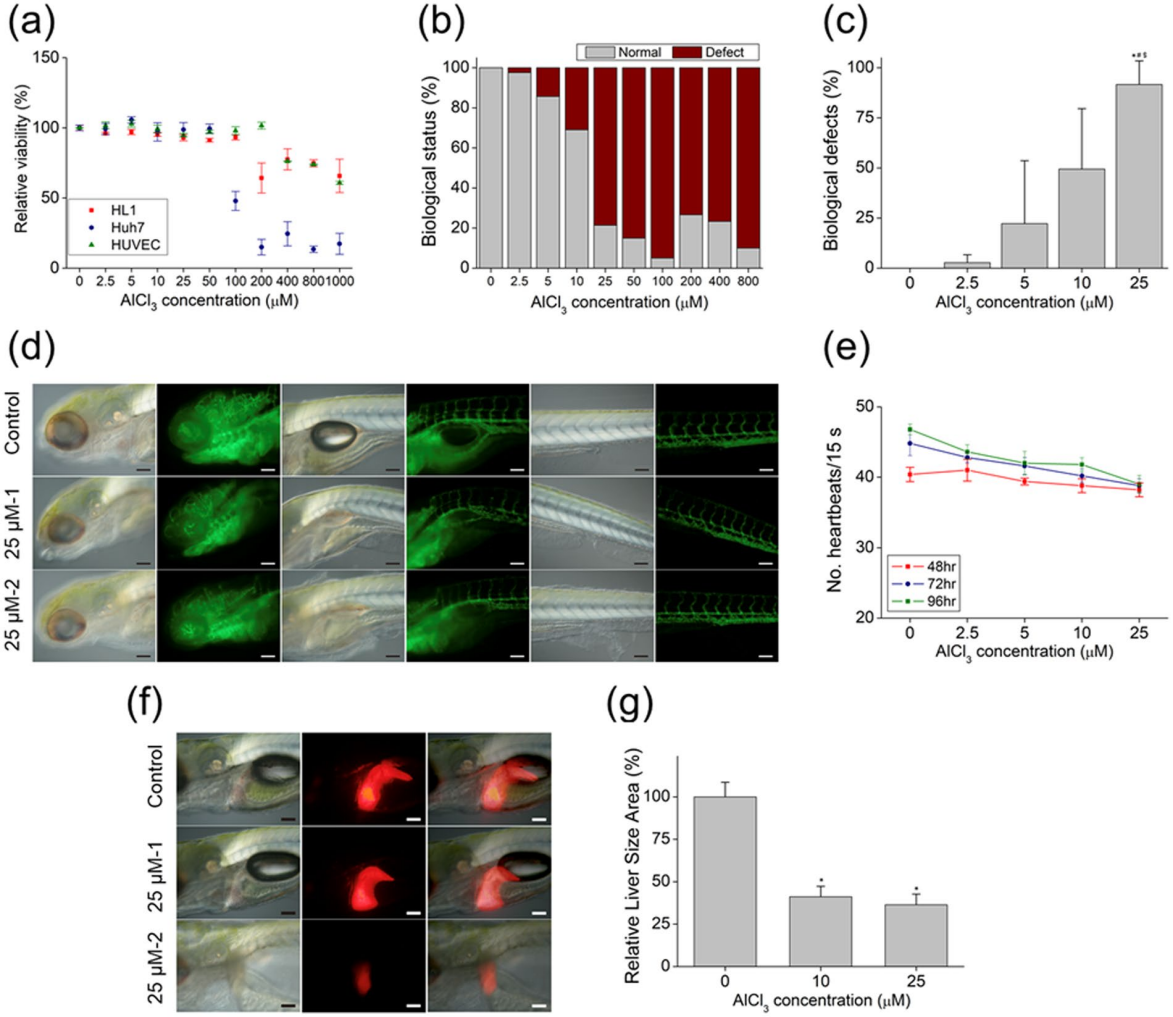
Biological effect of Al^3+^ *in vitro* and in a zebrafish model. (**a**) Viability of HL-1 and Huh7 cells and HUVECs after treatment with indicated ion solutions for 12 h. (**b**) Biological status of embryos (normal or defective) at 6 hpf after treatment with indicated ion solutions. (**c**) Biological defects in embryo at 6 hpf after treatment with indicated ion solutions. (**d**) Representative images of ion-treated zebrafish embryos used to determine heart rate. (**e**) Mean heart rate of zebrafish embryos following 48-, 72-, and 96-h treatments with different concentrations of Al^3+^. (**f**) Representative images of ion-treated zebrafish embryo used to determine liver size at 96 and 168 hpf. (**e**) Mean liver size of zebrafish embryos following 96-and 168-h treatments with indicated concentrations of Al^3+^. *, #, &, and +, Significant difference vs. 0, 2.5, and 5 μM of Al^3+^, respectively.

The viability of HL-1 and Huh7 cells and HUVECs was decreased to 64.25% ± 10.72%, 47.88% ± 6.74%, and 76.19% ± 0.14% at AlCl_3_ concentrations of 200, 100, and 400 μM, respectively (Fig. 5a). In zebrafish embryos, nervous system defects began to appear in the presence of 2.5 μM AlCl_3_, and reached 100% at concentrations above 25 μM (Fig. 5b,c and Supplementary Table 1).

The effect of Al ions on the circulatory system was evaluated using the Tg(flk1:EGFP) and Tg(cmlc2:EGFP) zebrafish lines. Embryos treated with 25 μM of AlCl_3_ showed a bent trunk phenotype; moreover, the overall structure of the tissue was disorganized, resulting in an abnormal morphology (Fig. 5d). Although there were no heart deformities, heart rate decreased in a concentration- and treatment time-dependent manner (Fig. 5e, and Supplementary Movies 3–6). These results indicate that, even at low concentrations, Al induces vascular defects and reduces cardiac function.

To examine the effects of AlCl_3_ on liver development, Tg(lfabp:DsRed) zebrafish expressing RFP in the liver were treated with varying concentrations of AlCl_3_. Concentrations of 10 and 25 μM decreased liver size to 36.43% ± 3.63% and 41.5% ± 3.55%, respectively, at 96 hpf (Fig. 5g), which was confirmed by microscopic examination (Fig. 5f). Evaluation of Al on neuronal growth was also performed *in vivo* using the Tg(GFAP:EGFP) and Tg(olig2:DsRed) transformation zebrafish model. However, Al had no effect on neurogenesis (Supplementary Fig. 1). Previous cell viability studies have shown that AlCl_3_ induces toxicity at concentrations of 800 μM or higher^33^; however, our *in vivo* results show that biological defects appear at 5 μM AlCl_3_, which is almost 10 times lower.

## Conclusions

Biocompatibility assessments of biodegradable Mg alloys have mostly used osteoblasts, macrophages, fibroblasts, and endothelial cells^32,34–36^. This has yielded variable rates of cell viability; moreover, some cell types are much more resilient to toxic ions than living tissues. It is presumed that *in vivo* studies provide more reliable data on biocompatibility. However, the high cost and longer observation time required have limited such evaluations in the development of biodegradable Mg alloys. To this end, transgenic zebrafish are a useful *in vivo* model that allows direct observation of developmental defects in specific organs caused by exposure to alloying elements^37,38^. Despite the hepatotoxicity of yttrium^39^ and the link between aluminum and Alzheimer’s disease^40^, these elements are often used for alloying to improve material strength and increase corrosion resistance. Studies have often demonstrated the safety of these alloys using cellular viability analyses and have emphasized that the amount of these elements are not substantial in the alloy system. However, the results of the present study clearly show that the *in vivo* biological responses to these toxic elements occur at significantly lower concentration than that observed in *in vitro* cellular assays. Utilization of zebrafish embryo allows fast, efficient, and accurate observation of *in vivo* toxicity of such alloy systems. This method can be further applied to test the extract media from the degrading alloys to observe the real-time toxicity of degrading particles.

## Methods

### Cell cultures

HL-1 cardiac muscle-like cells, Huh7 hepatocytes, and HUVECs were used for *in vitro* studies. HL-1 cells (gift from Dr. Craig Lygate, University of Oxford) were grown in Claycomb medium supplemented with 10% fetal bovine serum (FBS), 1% penicillin-streptomycin, and 1% l-glutamine in a fibronectin/gelatin pre-coated culture dish. Huh7 cells (gift from Dr. Leanne Hodson, University of Oxford) were cultured in Dulbecco’s Modified Eagle’s Medium with 10% FBS and 1% penicillin-streptomycin. HUVECs (Sigma-Aldrich, St. Louis, MO, USA) were maintained in endothelial cell growth medium. All cells were cultured under standard conditions (37 °C, 5% CO_2_).

### Cell viability assay

Cell viability was evaluated using a water-soluble tetrazolium salt (WST)-1 colorimetric assay (Abcam, Cambridge, MA, USA). Huh7 cells and HUVECs were seeded at a density of 1 × 10^4^ cells/cm^2^ in a 96-well plate. HL-1 cells were seeded at a density of 1 × 10^4^ cells/cm^2^ in a fibronectin/gelatin pre-coated 96-well plate. After 24 h, WST-1 reagent was added to the cultures, followed by incubation for an additional 2 h. Enzymatic activity was measured at 450 nm on a spectrophotometer. Data were calibrated to the absorbance value of cell-free medium and then normalized to the value for cells cultured without ion treatment.

### Chemical treatment of zebrafish embryos

MgCl_2_, ZnCl_2_, CaCl_2_, YCl_3_, and AlCl_3_ were dissolved with distilled water and diluted with zebrafish embryo medium in 6-well plates. A total of 10–15 zebrafish embryos were treated at 6 hpf with MgCl_2_, ZnCl_2_, CaCl_2_, YCl_3_, and AlCl_3_ solutions at various concentrations. The embryos were observed with Stemi 2000, Imager Z1, and Axioskop (Carl Zeiss, Oberkochen, Germany) and MZFL III, S6D, and DMI6000B (Leica, Wetzlar, Germany) microscope systems. The assay was repeated three times.

### Zebrafish models

We used Tg(flk1:EGFP), Tg(cmlc2:EGFP), Tg(lfabp:DsRed), Tg(GFAP:EGFP), and Tg(olig2:DsRed) zebrafish lines and wild-type (standard AB strain) zebrafish were used for *in vivo* experiments. Each zebrafish line was maintained at 28 °C under a 14:10-h light/dark cycle. Embryos were collected following natural mating of the parent zebrafish.

### Zebrafish embryo developmental toxicity test

Developmental toxicity was evaluated using embryos at 6 hpf with two or three replicates per exposure group and 10–15 fertilized eggs per replicate. The test solutions were refreshed once every 2 days after the initial exposure. During the experimental period, the number of unhatched eggs and dead and abnormal larvae were recorded to calculate the rate of biological defects [biological defects = (unhatched egg + total dead larvae + surviving abnormal larvae)/(initial count)]. Developmental toxicity was monitored by microscopy after exposure to YCl_3_ and AlCl_3_ at approximately 48, 72, 96, 120, 144, and 168 hpf.

### Examination of heart function

Wild-type zebrafish served as the control group for heartbeat counts at 48, 72, and 96 hpf. The number of heartbeats in 15 s was counted in five zebrafish from each group.

### Statistical analysis

Sigmoidal dose-response curves were generated for zebrafish embryos exposed to MgCl_2_, ZnCl_2_, CaCl_2_, YCl_3_, and AlCl_3_. The combined effects of the mixtures were evaluated with a post hoc Student–Newman–Keuls test following one-way analysis of variance using SigmaPlot v.12.5 software (Systat, San Jose, CA, USA). Data are shown as the mean and ± standard error of mean. Statistical significance was set as *p* < 0.05.

## Electronic supplementary material

Supplementary Information

Supplementary Movie 1. Heartbeat observation of Tg(cmlc2:EGFP) YCl3 control group at 72hpf

Supplementary Movie 2. Heartbeat observation of Tg(cmlc2:EGFP) group treated with 25μM of YCl3 at 72hpf

Supplementary Movie 3. Heartbeat observation of Tg(cmlc2:EGFP) AlCl3 control group at 72hpf

Supplementary Movie 4. Heartbeat observation of Tg(cmlc2:EGFP) group treated with 25μM of AlCl3 at 72hpf

Supplementary Movie 5. Heartbeat observation of Tg(cmlc2:EGFP) AlCl3 control group at 148hpf

Supplementary Movie 6. Heartbeat observation of Tg(cmlc2:EGFP) group treated with 25μM of AlCl3 at 148hpf
