# Supplementary material for: Transgenic zebrafish model for quantification and visualization of tissue toxicity caused by alloying elements in newly developed biodegradable metal
Source: Sci Rep. 2018 Sep 14;8:13818. doi: 10.1038/s41598-018-32313-5 (PMC6138638; doi:10.1038/s41598-018-32313-5)
Supplement: Supplementary file 1 — Supplementary Information [file 41598_2018_32313_MOESM1_ESM.docx]

**Transgenic zebrafish model for quantification and visualization of tissue toxicity caused by alloying elements in newly developed biodegradable metal**

Hyung-Seop Han^1,2‡^, Gun Hyuk Jang^2,3‡^, Indong Jun^1‡^, Hyunseon Seo^2^, Jimin Park^4^, Sion Glyn-Jones^1^, Hyun-Kwang Seok^2,5^, Kwan Hyi Lee^2,5^, Diego Mantovani^6^, Yu-Chan Kim^2,5*^, and James R. Edwards^1*^

^1^ Botnar Research Centre, Nuffield Department of Orthopaedics, Rheumatology and Musculoskeletal Sciences, University of Oxford, Oxford OX3 7LD, U.K

^2^ Center for Biomaterials, Korea Institute of Science and Technology, Seoul 02792, Republic of Korea

^3^ NuclixBio, Seoul 08380, Republic of Korea

^4^ Department of Materials Science and Engineering, Massachusetts Institute of Technology, Cambridge, Massachusetts 02139, USA.

^5^ Division of Bio-Medical Science and Technology, KIST School, Korea University of Science and Technology, Seoul 02792, Republic of Korea

^6^ Laboratory for Biomaterials and Bioengineering, CRC-I, Dept. Min-Met-Materials Engineering & CHU de Québec Research Center, Laval University, Quebec City, Canada

*Corresponding authors: chany@kist.re.kr (Y.-C.K) and james.edwards@ndorms.ox.ac.uk (J.R.E.)

^‡^These authors contributed equally to this work.

|  | | Live embryos (%) | Abnormal development (%) | Not hatched (%) | Cardiac edema (%) | Shortage (%) | Bent tail (%) |
| --- | --- | --- | --- | --- | --- | --- | --- |
| Control | | 100 | - | - | - | - | - |
| MgCl_2_ | 8 mM | 96.67 ± 4.71 | - | - | - | - | - |
|  | 16 mM | 100 | - | - | - | - | - |
|  | 32 mM | 70.63 ± 7.12 | 2.38 ± 3.37 | - | 2.22 ± 3.14 | - | - |
|  | 48 mM | 26.67 ± 37.71 | 11.11 ± 3.14 | - | - | - | 2.22 ± 3.14 |
|  | 64 mM | 2.22 ± 3.14 | - | - | - | - | - |
| ZnCl_2_ | 32 μM | 100 | 15.00 ± 16.50 | - | - | - | 13.33 ± 18.86 |
|  | 64 μM | 97.78 ± 3.14 | 27.22 ± 31.34 | - | - | - | 38.33 ± 37.04 |
|  | 125 μM | 88.89 ± 3.14 | 2.22 ± 12.57 | - | - | - | 31.11 ± 3.14 |
|  | 250 μM | 20.00 ± 18.85 | 6.66 ± 9.42 | - | - | - | 8.88 ± 8.31 |
|  | 500 μM | 0 | - | - | - | - | - |
| CaCl_2_ | 64 mM | 97.77 ± 3.14 | 26.66 ± 11.33 | - | - | - | - |
|  | 96 mM | 62.22 ± 44.00 | 55.56 ± 44.00 | - | - | - | - |
|  | 128 mM | 0 | - | - | - | - | - |
| YCl_3_ | 5 μM | 91.11 ± 8.31 | - | - | 13.89 ± 14.16 | - | 21.66 ± 16.50 |
|  | 10 μM | 70.56 ± 15.89 | - | - | 9.44 ± 2.83 | - | 22.22 ± 15.71 |
|  | 25 μM | 54.44 ± 38.52 | - | - | 15.00 ± 10.80 | - | 12.78 ± 10.21 |
|  | 50 μM | 43.89 ± 31.63 | - | - | 32.78 ± 30.44 | - | 13.89 ± 14.16 |
| AlCl_3_ | 2.5 μM | 97.22 ± 4.81 | - | - | - | - | - |
|  | 5 μM | 86.67 ± 18.85 | - | 20.00 ± 12.57 | 8.89 ± 12.57 | - | 32.22 ± 12.86 |
|  | 10 μM | 70.56 ± 26.40 | - | 17.78 ± 8.31 | 4.44 ± 6.28 | - | 27.22 ± 11.57 |
|  | 25 μM | 23.89 ± 24.96 | - | 15.55 ± 13.70 | 4.44 ± 6.28 | 17.22 ± 12.20 | 11.11 ± 8.31 |

**Table S1. Recorded biological defects for Mg, Zn, Ca, Y, Al ions**

|  | | 48 hpf | 72 hpf | 96 hpf |
| --- | --- | --- | --- | --- |
| Control | | 38.55 ± 1.88 | 42.90 ± 2.80 | 42.60 ± 4.29 |
| MgCl_2_ | 32 mM | - | 36.40 ± 1.02 | 36.00 ± 1.95 |
|  | 48 mM | - | 35.40 ± 0.80 | 35.00 ± 1.26 |
|  | 64 mM | - | 35.00 ± 1.67 | 33.40 ± 1.36 |
| ZnCl_2_ | 125 μM | - | 36.80 ± 3.54 | 34.40 ± 1.02 |
|  | 250 μM | - | 35.80 ± 0.75 | 34.60 ± 1.20 |
|  | 500 μM | - | 31.60 ± 3.88 | 36.60 ± 1.02 |
| CaCl_2_ | 64 mM | - | 35.20 ± 1.60 | 34.00 ± 0.63 |
|  | 96 mM | - | 35.20 ± 1.33 | 33.60 ± 1.36 |
|  | 128 mM | - | 28.60 ± 1.62 | 26.60 ± 2.06 |
| YCl_3_ | 5 μM | 33.60 ± 0.80 | 40.40 ± 1.02 | 41.00 ± 0.89 |
|  | 10 μM | 32.60 ± 0.49 | 39.40 ± 1.74 | 39.20 ± 0.98 |
|  | 25 μM | 31.80 ± 0.98 | 39.60 ± 1.62 | 39.00 ±0.89 |
|  | 50 μM | 25.40 ± 1.96 | 24.80 ± 0.75 | 27.00 ± 1.67 |
| AlCl_3_ | 2.5 μM | 41.00 ± 1.55 | 42.80 ± 0.75 | 43.60 ± 1.02 |
|  | 5 μM | 39.40 ± 0.49 | 41.60 ± 1.20 | 42.00 ± 1.67 |
|  | 10 μM | 38.80 ± 0.98 | 40.20 ± 0.98 | 41.80 ± 0.98 |
|  | 25 μM | 38.20 ±0.98 | 38.80 ± 0.98 | 39.00 ± 1.26 |

**Table S2. Heartrate per 15 second after treatment with Mg, Zn, Ca, Y, Al ions 48, 72 and 96 hpf**


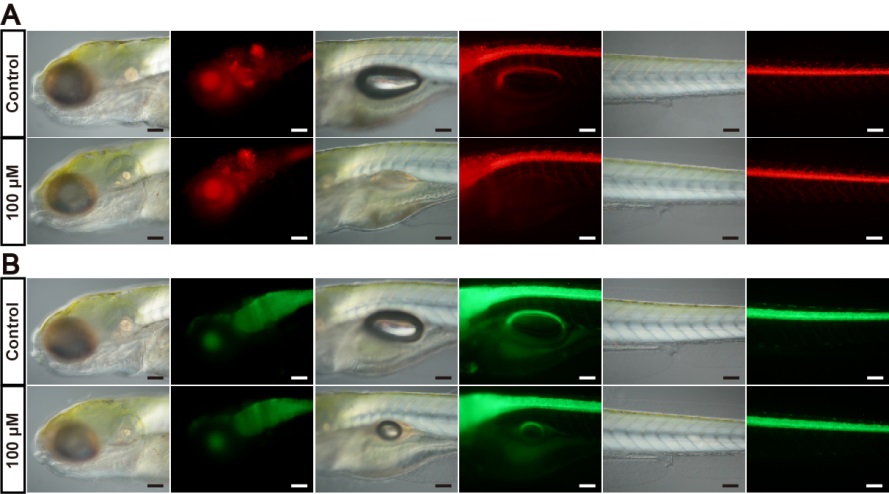


**Figure S1.** Evaluation of AlCl_3_ on neuronal growth A) 144hpf result of Tg(olig2:DsRed) zebrafish treated with 100 uM of AlCl_3_. B) 144hpf result of Tg(GFAP:EGFP) zebrafish treated with 100 uM of AlCl_3_.

Supplementary Movie 1. Heartbeat observation of Tg(cmlc2:EGFP) YCl_3_ control group at 72hpf

Supplementary Movie 2. Heartbeat observation of Tg(cmlc2:EGFP) group treated with 25μM of YCl_3_ at 72hpf

Supplementary Movie 3. Heartbeat observation of Tg(cmlc2:EGFP) AlCl_3_ control group at 72hpf

Supplementary Movie 4. Heartbeat observation of Tg(cmlc2:EGFP) group treated with 25μM of AlCl_3_ at 72hpf

Supplementary Movie 5. Heartbeat observation of Tg(cmlc2:EGFP) AlCl_3_ control group at 148hpf

Supplementary Movie 6. Heartbeat observation of Tg(cmlc2:EGFP) group treated with 25μM of AlCl_3_ at 148hpf
